# Prevalence, associated factors, and comorbidities of tinnitus in adolescents

**DOI:** 10.1371/journal.pone.0236723

**Published:** 2020-07-31

**Authors:** Jihye Rhee, Dongwook Lee, Myung Whan Suh, Jun Ho Lee, Yun-Chul Hong, Seung Ha Oh, Moo Kyun Park

**Affiliations:** 1 Department of Otorhinolaryngology, Veterans Health Service Medical Center, Seoul, Korea; 2 Department of Preventive Medicine, Seoul National University College of Medicine, Seoul, Korea; 3 Department of Otorhinolaryngology-Head and Neck Surgery, Seoul National University Hospital, Seoul National University College of Medicine, Seoul, Korea; 4 Sensory Organ Research Institute, Seoul National University Medical Research Center, Seoul, Republic of Korea; Korea University Medical College, REPUBLIC OF KOREA

## Abstract

The aim of this study was to investigate the prevalence of tinnitus among adolescents, and the factors known or hypothesized to be associated with tinnitus. Based on random sampling of school types and regions, a nationwide hearing survey of the first-year middle and high school students of South Korea was performed. The subjects underwent an otologic examination followed by pure tone audiometry up to 8 kHz. Questionnaires about the factors associated with hearing and tinnitus were completed by the students and their parents. Among the 1,593 subjects who completed the questionnaire and underwent pure tone audiometry, the prevalence of tinnitus was 46.0% and that of severe tinnitus was 9.1%. Tinnitus was associated with age, female gender, history of ear infection and sinusitis, leisure noise exposure due to karaoke and local-area-network gaming, alcohol consumption, and cigarette smoking. Noticeable hearing loss was not detected but participants with tinnitus complained of difficulty with sound localization, hearing in noise, and verbal working memory and were more susceptible to fatigue. The subjects with tinnitus also suffered more physical and mental health problems than did those without tinnitus. Thus, protection of the ears from noise and appropriate counseling should be considered for adolescents with tinnitus.

## Introduction

Tinnitus is defined as the sensation of sound without external stimulation. It is one of the common symptoms in auditory system. [1] Most people suffer from tinnitus at least once in their life. About 30% of the general population experience tinnitus and about 10–15% have chronic tinnitus that requires medical evaluation. About 6% of tinnitus cases are considered severe. [2] However, tinnitus in adolescents has not been investigated extensively. The prevalence of tinnitus in adolescents varies from 4.7% to 74.9% according to the definition of tinnitus and the study design and population. [3] Because adolescents seldom complain of tinnitus spontaneously, most studies have been retrospective or not nationwide. Therefore, the above-mentioned prevalence of tinnitus among adolescents could be an underestimate.

The factors associated with, and the comorbidities of, tinnitus in adolescents are unclear. Tinnitus is related to hearing loss or noise exposure. [4, 5] A hearing examination is crucial for the evaluation of tinnitus. Tinnitus could be related to auditory neuroplasticity after noise exposure. However, the association between hearing loss and noise exposure is unclear. Tinnitus is significantly associated with a poor quality of life, sleep disturbance, absence from work, and learning difficulty. [6–8] Moreover, tinnitus can degrade the understanding of speech in noise [9] and sound localization. [10] Patients with tinnitus show a variety of somatization symptoms and tinnitus could be related to psycho-emotional problems such as anxiety and depression. [8] Knowledge of the associated factors and comorbidities of tinnitus is important for providing appropriate management to patients.

We investigated the prevalence, severity, associated factors, and comorbid conditions of tinnitus in a nationwide hearing survey of middle- and high-school students by means of pure-tone audiometry (PTA), a hearing performance (HP) questionnaire, and a history of leisure noise exposure (LNE), and by evaluating socioeconomic, physical health, and mental health factors.

## Materials and methods

### Study design and subjects

We designed a nationwide cross-sectional survey of first-year middle (12–13 years old) and high (15–16 years old) school students based on random sampling of single-gender, coeducational, general, and vocational schools in eight metropolitan and eight suburban regions. The sampling protocol was described previously. [11] After a pilot study, which showed 50% agreement on the part of schools to participate in the survey, we listed 248 from 5,587 schools in Korea. The number of participants in each school was 25, based on the average number of students per class. The target class was carefully selected to be representative of the characteristics of the school. Two types of written informed consent were provided individually, to the students and their parents. Only the students their parents, who had both agreed to participate in the survey, were evaluated. Among the participants, those who had conductive hearing loss and/or any abnormalities in an otologic examination were excluded. The study was approved by the Institutional Review Board of Seoul National University Hospital (No. 1604-086-755). It was conducted in accordance with the principles that have their origin in the Declaration of Helsinki.

### Data collection

As tinnitus is a subjective symptom, no objective instruments are available for its measurement. Therefore, questionnaires were chosen with reference to previously reported epidemiological research on tinnitus. [12, 13] The questionnaires provided to the participants included two questions regarding tinnitus: (1) the presence or absence of tinnitus and (2) the degree of tinnitus. Those participants who responded that they had ever heard a sound were instructed to answer the second question about the degree of tinnitus, the possible responses to which were: “No problem”, “I am annoyed and bothered”, and “I find it hard to sleep.” The participants were asked to choose only one of the responses. Based on the responses, we defined the following three groups: (1) “normal” for students without tinnitus, (2) “mild tinnitus” for students who had tinnitus, and chose “no problem” as the severity, and (3) “severe tinnitus” for students who were annoyed, bothered, or had sleep problems due to their tinnitus.

A part of the questionnaire inquired about the risk of LNE. The usage of personal listening devices, presence or absence of the experience of conflict with others due to the volume of a listening device, and the time and frequency of usage of leisure facilities (such as local area network (LAN) gaming centers, karaoke rooms, and concert auditoriums).

The participants also responded to four questions about auditory behavior. They were about their hearing in a noisy environment (“Have difficulty hearing or understanding in a noisy environment”), sound localization (“Have trouble finding the sound direction”), verbal working memory (“Have difficulty memorizing by listening to verbal orders”), and fatigue (“Easily feel tired”). The participants responded to the questions by assigning a score of 1 (worst) to 5 (best).

The subjects self-rated their academic performance to explore the association between educational attainment and tinnitus. The subjects responded to the following question: “Please rate your grades for the past year” using a five-point Likert scale (1, highest; 2, high; 3, middle; 4, low; and 5, lowest).

The subjects’ health status, medical history of themselves and their families, alcohol consumption, and smoking were also assessed. The questionnaire was based on Forms 1–2 and 1–3 of the Enforcement Rule of Health Screening at School, according to the School Health Act of South Korea. The health condition questionnaire inquired about general symptoms, respiratory system symptoms, circulatory system symptoms, digestive system symptoms, mental health, hematopoietic system symptoms, and about lifestyle factors such as diet, personal hygiene, exercise, safety, Internet use, home and school life, drug use, and sexuality; the possible responses were yes or no.

### Audiological evaluation

PTA was performed by audiometer (AD299b, Interacoustics, Assen, Denmark) in a sound-proof booth in a mobile vehicle. Four experienced audiologists conducted PTA testing at 0.5, 1, 2, 3, 4, 6, and 8 kHz. The hearing threshold was determined as the lowest sound level that was responded to correctly for 50% of stimuli. For frequencies with hearing impairment of ≥ 25 dB, the bone conduction threshold was evaluated to rule out the possibility of conductive hearing loss. High frequencies were defined as 3, 4, 6, and 8 kHz and speech frequencies were defined as 0.5, 1, and 2 kHz. Before the PTA, an otologic examination was performed to remove materials (such as cerumen and foreign bodies) that could affect hearing.

### Statistical analyses

We estimated the prevalence of tinnitus among Korean adolescents. The unweighted frequencies and weighted prevalences were used because of the multi-stage complex sampling design of the study. According to characteristics of participants, the prevalence of any tinnitus (mild or tinnitus) and severe tinnitus was calculated, and univariate logistic regression analyses were performed. We also estimated the arithmetic mean and standard error of the pure-tone threshold of each frequency, the pure-tone averages of speech and high frequencies, according to the severity of tinnitus. Differences among means were tested by analysis of variance. A multivariate logistic regression was performed to investigate the association of LNE with severe tinnitus, with adjustment variables for the following potential confounders: age, gender, household income, obesity, alcohol consumption, history of otitis, and history of sinusitis with reference to the results of our analyses and a previous report. [14] Finally, the mean values of HP (hearing in a noisy environment, sound localization, verbal working memory, and fatigue) were compared according to tinnitus severity. Multivariate linear regression analyses were performed to assess the differences in mean values by tinnitus severity with adjustment for potential confounders. Additionally, to investigate the associations between academic performance and hearing status, the mean subjective academic performance scores and 95% confidence intervals were calculated and subjected to multivariate linear regression analysis according to tinnitus status. Moreover, we investigated the prevalence of tinnitus according to the responses to the physical health, mental health, and lifestyle items in the questionnaire by chi-squared test with Bonferroni correction for multiple comparisons (corrected significance level, *P* < 0.0009). Statistical analyses were performed using the SAS SURVEYFREQ, SAS SURVEYLOGISTIC, SAS SURVEYREG, and SAS FREQ procedures (ver. 9.4; SAS Institute, Inc., Cary, NC), and statistical significance was defined as a two-sided *P* ≤ 0.05.

## Results

**Fig 1:**
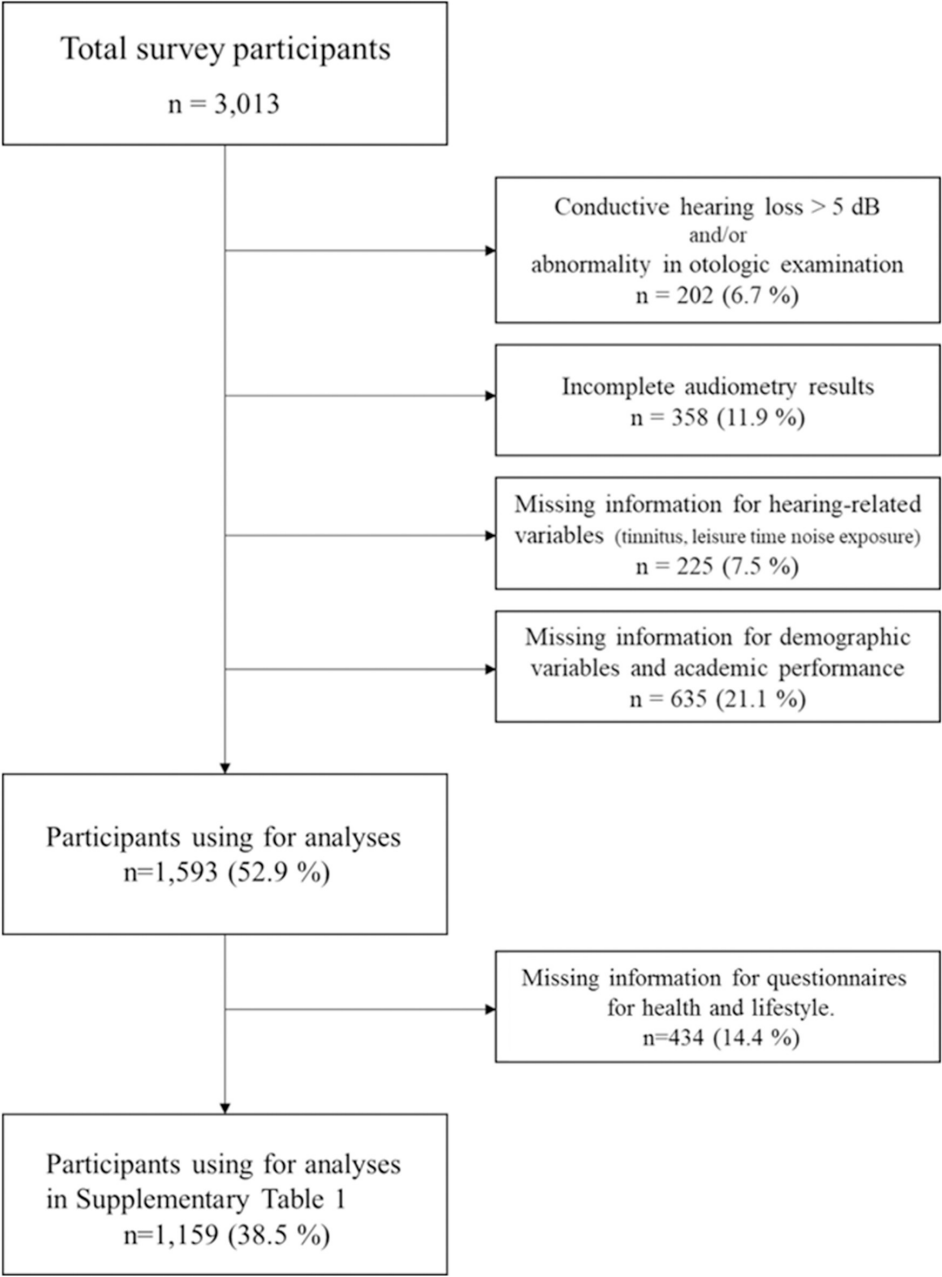
Flow chart of selection of the subjects.

Among the 248 schools, 109 (3,013 students) agreed to participate in the study. After excluding participants with incomplete answers (n = 860) and missing PTA data (n = 358), 1,795 participants were considered for inclusion. A further 202 subjects were excluded because of conductive hearing loss detected by PTA and/or abnormalities identified by otologic examination (*e*.*g*., perforation or retraction of tympanic membrane, middle ear effusion, or ear anomalies). Finally, 1,593 subjects were enrolled in the study. Additionally, analyses for the associations between questionnaires for health and lifestyle and tinnitus were performed after excluding missing variables for the questionnaires (n = 434) (Fig 1).

Among the 1,593 students, 46.0% (n = 755) responded that they had suffered tinnitus in the past year. The prevalence of severe tinnitus that induced some degree of discomfort was 9.1% (n = 154). Severe tinnitus was more common in high school students (OR, 1.52; 95% CI, 1.03–2.23) and female students (OR, 2.47; 95% CI, 1.53–3.99) than in the others. Students who had consumed alcohol or smoked cigarettes had a higher prevalence of tinnitus. A history of sinusitis and of ear infection were also associated with tinnitus. Also, a history of ear infection was significantly associated with severe tinnitus causing discomfort. Among the risk factors, household income and BMI were not associated with the prevalence of tinnitus (Table 1). The hearing threshold of each frequency and the average thresholds of speech frequencies and high frequencies were not worse in the mild or severe tinnitus group than in the normal group (Table 2).

**Table 1:** Prevalence of tinnitus according to the participants’ characteristics.

	Total	Any tinnitus		Severe tinnitus	
	n (%)^a^	n (%)^b^	OR (95% CI)	n (%)	OR (95% CI)
**Total**	1593 (100)	755 (46.0)		154 (9.1)	
**School Level**					
Middle school	848 (51.1)	326 (37.2)	1 (Ref)	57 (7.5)	1 (Ref)
High school	745 (48.9)	429 (55.1)	2.07 (1.73–2.48)**	97 (10.9)	1.52 (1.03–2.23)*
**Gender**					
Male	804 (51.8)	315 (38.9)	1 (Ref)	57 (5.6)	1 (Ref)
Female	789 (48.2)	440 (53.7)	1.82 (1.44–2.31)**	97 (12.9)	2.47 (1.53–3.99)**
**Household income per month, US$**					
< 2,000	259 (13.5)	124 (52.1)	1 (Ref)	25 (11.6)	1 (Ref)
2,000–3,000	310 (19.0)	148 (46.4)	0.80 (0.58–1.09)	32 (9.7)	0.83 (0.48–1.41)
3,000–4,000	402 (25.2)	191 (44.6)	0.74 (0.52–1.06)	35 (9.1)	0.76 (0.39–1.49)
> 4,000	622 (42.3)	292 (44.7)	0.74 (0.55–1.02)	62 (8.1)	0.68 (0.40–1.16)
**BMI, kg/m**^**2**^					
< 25	1349 (84.5)	633 (45.7)	1 (Ref)	131 (9.5)	1 (Ref)
≥ 25	244 (15.5)	122 (47.9)	1.09 (0.81–1.48)	23 (6.9)	0.71 (0.49–1.01)
**Smoking status**					
Never	1453 (90.7)	684 (45.0)	1 (Ref)	134 (8.5)	1 (Ref)
Ever	140 (9.3)	71 (55.7)	1.53 (1.11–2.12)*	20 (15.0)	1.89 (0.88–4.03)
**Alcohol consumption**					
Never	1282 (81.4)	568 (43.4)	1 (Ref)	113 (8.7)	1 (Ref)
Ever	311 (18.6)	187 (57.6)	1.77 (1.30–2.42)**	41 (11.0)	1.30 (0.83–2.04)
**History of sinusitis**					
No	1438 (89.7)	673 (45.1)	1 (Ref)	131 (8.8)	1 (Ref)
Yes	155 (10.3)	82 (53.7)	1.41 (1.13–1.76)*	23 (12.3)	1.46 (0.96–2.20)
**History of ear infection**					
No	1435 (90.4)	662 (44.5)	1 (Ref)	128 (8.0)	1 (Ref)
Yes	158 (9.6)	93 (60.0)	1.87 (1.32–2.66)**	26 (19.8)	2.84 (1.46–5.53)*

^a^ Unweighted frequencies and weighted percentages

^b^ Unweighted frequencies and weighted row percentages.

* *P* < 0.05

** *P* < 0.001

OR, odds ratio; CI, confidence interval; BMI, body mass index; Ref, reference

**Table 2:** Results of pure-tone audiometry according to tinnitus symptom.

		Right (mean ± SE)				Left (mean ± SE)			
		Normal (n = 838)	Mild tinnitus (n = 601)	Severe tinnitus (n = 154)	*P*^a^	Normal (n = 838)	Mild tinnitus (n = 601)	Severe tinnitus (n = 154)	*P*^a^
**Average**	**Speech**^b^	8.7 ± 0.2	8.8 ± 0.2	8.6 ± 0.4	0.707	7.4 ± 0.2	7.6 ± 0.2	7.3 ± 0.6	0.459
	**High**^c^	4.8 ± 0.1	4.9 ± 0.1	4.5 ± 0.3	0.232	4.3 ± 0.1	4.5 ± 0.1	4.2 ± 0.4	0.371
**Frequency**	**0.5 kHz**	10.9 ± 0.4	10.3 ± 0.3	10.8 ± 0.5	0.177	9.9 ± 0.3	10.2 ± 0.4	10.5 ± 0.6	0.437
	**1 kHz**	8.0 ± 0.2	8.4 ± 0.3	7.8 ± 0.4	0.263	6.4 ± 0.2	6.5 ± 0.3	6.0 ± 0.6	0.478
	**2 kHz**	7.2 ± 0.2	7.8 ± 0.2	7.1 ± 0.5	**0.041**	5.9 ± 0.2	6.2 ± 0.2	5.5 ± 0.6	0.169
	**3 kHz**	6.8 ± 0.2	6.8 ± 0.2	6.4 ± 0.5	0.642	5.8 ± 0.2	6.4 ± 0.2	5.9 ± 0.4	0.164
	**4 kHz**	7.9 ± 0.3	7.9 ± 0.3	6.8 ± 0.5	0.118	6.7 ± 0.2	6.7 ± 0.3	6.4 ± 0.9	0.828
	**6 kHz**	2.1 ± 0.1	2.1 ± 0.1	2.2 ± 0.2	0.586	2.1 ± 0.1	2.2 ± 0.1	2.0 ± 0.2	0.439
	**8 kHz**	2.6 ± 0.1	2.6 ± 0.1	2.4 ± 0.2	0.571	2.6 ± 0.1	2.7 ± 0.1	2.5 ± 0.2	0.687

^a^ Analysis of variance was performed because of the complex structure of the survey.

^b^ Pure tone averages of 0.5, 1, and 2 kHz

^c^ Pure tone averages of 3, 4, 6, and 8 kHz

Among the LNE, usage of LAN gaming centers and usage of karaoke facilities were associated with severe tinnitus after adjusting for potential confounders (Table 3). Severe tinnitus was significantly associated with usage of LAN gaming centers (OR, 1.52; 95% CI, 1.02–2.24), and karaoke facilities (OR, 1.72; 95% CI, 1.01–2.94).

**Table 3:** Multivariate logistic regression of the association between leisure-time noise exposure and tinnitus.

	Total	Severe tinnitus	Unadjusted model	Adjusted model^a^
	n (%)^b^	n (%)^c^	OR (95% CI)	OR (95% CI)
**Personal listening device use**				
No	148 (10.1)	12 (6.7)	1 (Ref)	1 (Ref)
Yes	1428 (89.9)	142 (9.5)	1.51 (0.86–2.66)	1.23 (0.68–2.21)
**Request to turn down the volume**				
Not applicable	148 (10.1)	12 (6.7)	1 (Ref)	1 (Ref)
Never experienced	237 (13.8)	33 (11.3)	0.69 (0.38–1.22)	0.83 (0.46–1.51)
Ever experienced	1191 (76.2)	109 (9.2)	1.26 (0.71–2.23)	1.14 (0.67–1.95)
**Usage of LAN gaming center in the past year**				
No	638 (40.1)	53 (8.7)	1 (Ref)	1 (Ref)
Yes	938 (59.9)	101 (9.5)	1.11 (0.74–1.65)	1.52 (1.02–2.24)*
**Usage of karaoke in the past year**				
No	386 (23.4)	24 (4.9)	1 (Ref)	1 (Ref)
Yes	1190 (76.6)	130 (10.5)	2.33 (1.29–4.21)*	1.72 (1.01–2.94)*
**Attendance at a concert in the past year**				
No	1372 (88.3)	133 (9.2)	1 (Ref)	1 (Ref)
Yes	204 (11.7)	21 (9.3)	1.02 (0.61–1.71)	0.74 (0.42–1.29)
**Attendance at a dance club in the past year**				
No	1572 (99.6)	154 (9.2)	1 (Ref)	1 (Ref)
Yes	4 (0.4)	0 (0)	-	-

^a^ Adjusted for age, gender, household income, obesity, smoking, alcohol consumption, history of otitis, and history of sinusitis

^b^ Unweighted frequencies and weighted percentages

^c^ Unweighted frequencies and weighted row percentages of severe tinnitus

* *P* < 0.05

OR, odds ratio; CI, confidence interval

**Fig 2:**
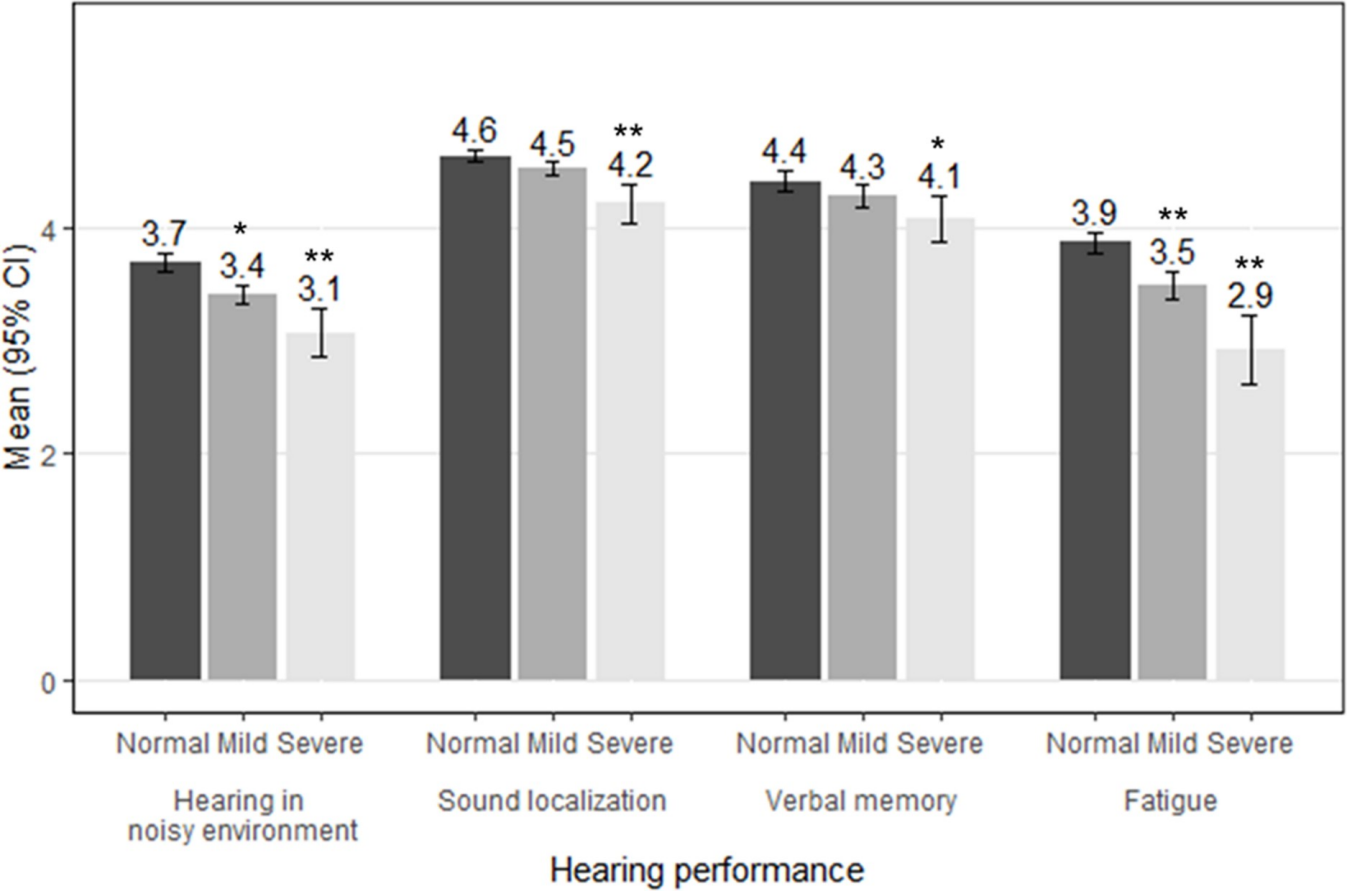
Logistic regression of the association between hearing performance and tinnitus severity.

The severity of tinnitus was significantly associated with a decreased HP (Fig 2). Compared with normal students, those with severe tinnitus had significantly decreased scores for hearing in a noisy environment (3.7 *vs*. 3.1, *P* < 0.001), sound localization (4.6 *vs*. 4.2, *P* < 0.001), verbal working memory (4.4 *vs*. 4.1, *P* = 0.004), and fatigue (3.9 *vs*. 2.9, *P* < 0.001).

**Fig 3:**
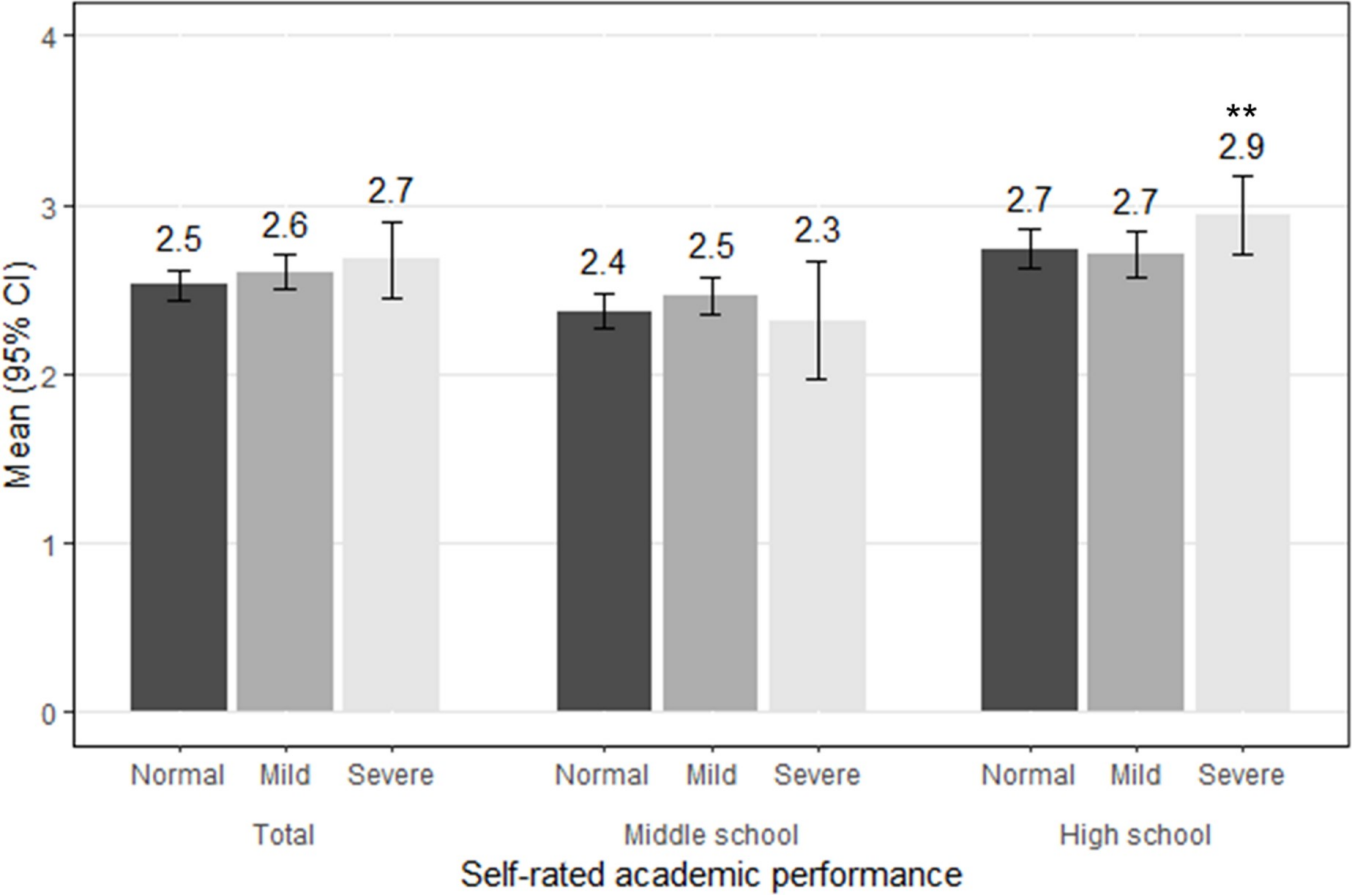
Subjective academic performance according to the severity of tinnitus. Subjective academic performance for the past year was assessed using the following question: “Please rate your grades for the past year (1, highest; 2, high; 3, middle; 4, low; and 5, lowest).” Multivariate linear regression analysis was performed to examine differences in subjective academic performance according to tinnitus status (mild and severe group) in comparison to the healthy control group. ** P < 0.001.

Severe tinnitus was associated with several physical and mental health factors: general condition, sore throat, gastrointestinal symptoms, depressive mood, hyperactivity, headache with throbbing pain, easy bruising, and menstrual pain (Table 4). Subjective academic performance was significantly associated with severe tinnitus only among high-school students (Fig 3).

**Table 4:** Prevalence of tinnitus according to physical health, mental health, and lifestyle factors. (the Enforcement Rule of Health Screening at School, according to the School Health Act of South Korea).

		No		Yes		
		n (%)	Severe Tinnitus n (%)	n (%)	Severe Tinnitus n (%)	*P*^a^
Condition	**Catch a cold easily**	**864 (74.6)**	**69 (8.0)**	**295 (25.5)**	**48 (16.3)**	** < 0.0001**
	**I lack strength and I get tired easily**	**807 (69.6)**	**57 (7.1)**	**352 (30.4)**	**60 (17.1)**	** < 0.0001**
	I think I am not healthy	1010 (87.1)	91 (9.0)	149 (12.9)	26 (17.5)	0.0014
Respiratory System	My Nose and eyes are itchy, I sneeze, and sometimes I have a runny nose.	808 (69.7)	71 (8.8)	351 (30.3)	46 (13.1)	0.0249
	I experience shortness of breath and I hear whistles from my chest.	1094 (94.4)	105 (9.6)	65 (5.6)	12 (18.5)	0.0212
	I have yellow sputum when I cough.	994 (85.8)	91 (9.2)	165 (14.2)	26 (15.8)	0.0091
	I usually have trouble breathing through my nose because my nose is blocked.	904 (78.0)	81 (9.0)	255 (22)	36 (14.1)	0.0158
	I am told that I often snore badly.	1087 (93.8)	106 (9.8)	72 (6.2)	11 (15.3)	0.1317
	**I often have fever and a sore throat.**	**1063 (91.7)**	**91 (8.6)**	**96 (8.3)**	**26 (27.1)**	** < 0.0001**
	There is a lump in my neck.	1118 (96.5)	108 (9.7)	41 (3.5)	9 (22.0)	0.0103
Circulatory System	I get out of breath, even after a little exercise, compared with others.	970 (83.7)	88 (9.1)	189 (16.3)	29 (15.3)	0.0088
	My complexion is bad and my heart pounds even when I am at rest	1139 (98.3)	115 (10.1)	20 (1.7)	2 (10.0)	0.9887
Digestive	**I have a burning feeling or pain in my stomach**	**862 (74.4)**	**55 (6.4)**	**297 (25.6)**	**62 (20.9)**	** < 0.0001**
	**I feel stuffy or full.**	**891 (76.9)**	**60 (6.7)**	**268 (23.1)**	**57 (21.3)**	** < 0.0001**
	**My lower belly is painful, or I often have diarrhea.**	**946 (81.6)**	**77 (8.1)**	**213 (18.4)**	**40 (18.8)**	** < 0.0001**
	**My stomach fells tight or bloated.**	**985 (85.0)**	**77 (7.8)**	**174 (15)**	**40 (23.0)**	** < 0.0001**
Mental Health	**I feel hopeless because I am sad or depressed.**	**1022 (88.2)**	**90 (8.8)**	**137 (11.8)**	**27 (19.7)**	** < 0.0001**
	**I often do not want to go to school.**	**951 (82.1)**	**80 (8.4)**	**208 (18)**	**37 (17.8)**	** < 0.0001**
	I have seriously considered suicide or attempted suicide.	1132 (97.7)	112 (9.9)	27 (2.3)	5 (18.5)	0.1415
	I often feel nervous as if I am going crazy.	1111 (95.9)	110 (9.9)	48 (4.1)	7 (14.6)	0.2918
	**I am not calm. I am too active, which can interfere with the activities of other children.**	**1075 (92.8)**	**98 (9.1)**	**84 (7.3)**	**19 (22.6)**	** < 0.0001**
Blood	I often have nosebleeds and the bleeding does not stop quickly if I am injured.	1097 (94.7)	106 (9.7)	62 (5.4)	11 (17.7)	0.0399
	**I get bruised easily.**	**998 (86.1)**	**84 (8.4)**	**161 (13.9)**	**33 (20.5)**	** < 0.0001**
Other Symptoms	**My headache or migraine is severe.**	**1006 (86.8)**	**79 (7.9)**	**153 (13.2)**	**38 (24.8)**	** < 0.0001**
	My ears are sore, or I have an ear discharge.	1129 (97.4)	109 (9.7)	30 (2.6)	8 (26.7)	0.0023
	My jaw joints are sore, or I cannot open my mouth well.	1109 (95.7)	109 (9.8)	50 (4.3)	8 (16)	0.1565
	**I have a throbbing pain in my neck/waist/knee etc.**	**968 (83.5)**	**77 (8.0)**	**191 (16.5)**	**40 (20.9)**	** < 0.0001**
	**(For girls) My menstrual cramps are severe.**	**473 (79.4)**	**43 (9.1)**	**123 (20.6)**	**28 (22.8)**	** < 0.0001**
Diet	I eat breakfast regularly.	382 (33)	43 (11.3)	777 (67)	74 (9.5)	0.3573
	I drink milk or dairy products every day.	465 (40.1)	55 (11.8)	694 (59.9)	62 (8.9)	0.1089
	I eat vegetables and fruit every day.	315 (27.2)	37 (11.8)	844 (72.8)	80 (9.5)	0.2543
	I rarely eat sweet or salty food.	894 (77.1)	93 (10.4)	265 (22.9)	24 (9.1)	0.5229
	I consume soft drinks, hamburgers, pizza, or snacks almost every day.	942 (81.3)	86 (9.1)	217 (18.7)	31 (14.3)	0.0230
	I skip meals or take drugs to lose weight.	1108 (95.6)	109 (9.8)	51 (4.4)	8 (15.7)	0.1752
Personal Hygiene	I wash my hands with soap before eating or after going out.	346 (29.9)	40 (11.6)	813 (70.2)	77 (9.5)	0.2799
	I brush my teeth more than twice a day.	80 (6.9)	5 (6.3)	1079 (93.1)	112 (10.4)	0.2368
Exercise	I exercise intensely at least three times a week.	650 (56.1)	75 (11.5)	509 (43.9)	42 (8.3)	0.0653
	I allocate study and exercise time well. I feel refreshed after sleeping.	677 (58.4)	83 (12.3)	482 (41.6)	34 (7.1)	0.0037
Safety	I wear a seat belt when I ride in a car.	404 (34.9)	38 (9.4)	755 (65.1)	79 (10.5)	0.5690
	I wear a helmet or protective gear when I use inline skates, roller blades, skateboards, bicycles, etc.	812 (70.1)	86 (10.6)	347 (29.9)	31 (8.9)	0.3910
Internet	I used the internet or play games more than 2 hours a day.	715 (61.7)	70 (9.8)	444 (38.3)	47 (10.6)	0.6621
	I often watch pornography or chat on adult sites.	1124 (97)	111 (9.9)	35 (3)	6 (17.1)	0.1599
Home & School Life	I have been bullied or isolated by my friends over the past year.	1134 (97.8)	111 (9.8)	25 (2.2)	6 (24)	0.0196
	I have a person to talk to when I have worries or problems.	209 (18)	23 (11)	950 (82)	94 (9.9)	0.6296
	I am worried about problems in my family.	1030 (88.9)	99 (9.6)	129 (11.1)	18 (14)	0.1228
	I have seriously considered running away from home during the past year.	1105 (95.3)	105 (9.5)	54 (4.7)	12 (22.2)	0.0024
	My safety is threatened by violence at home or school.	1153 (99.5)	116 (10.1)	6 (0.5)	1 (16.7)	0.5921
	I have carried a knife/club/nunchaku as a weapon.	1151 (99.3)	114 (9.9)	8 (0.7)	3 (37.5)	0.0098
	I have used marijuana or psychotropic drugs to hallucinate.	1157 (99.8)	117 (10.1)	2 (0.2)	0 (0)	0.6353
Sexuality	I worry about gender issues.	1142 (98.5)	114 (10)	17 (1.5)	3 (17.7)	0.2978
	I have been abused physically, mentally, and/or sexually.	1155 (99.7)	116 (10)	4 (0.4)	1 (25)	0.3216

^a^ Chi-squared tests were performed, and *p-*values in bold indicate significance after Bonferroni correction for multiple comparisons (*P* < 0.0009).

## Discussion

This was the first official nationwide hearing survey of adolescents in South Korea. Previous studies used the data of the Korea National Health and Nutrition Examination Survey [15–18] or were questionnaire-based [8, 19]. The National Health and Nutrition Examination Surveys of Korea and the United States collect comprehensive health data. But this information is not intended for a review of hearing or tinnitus in adolescents. Therefore, most studies lacked detailed information on noise exposure history and risk factors for hearing or tinnitus; also, the PTA testing did not include 8 kHz, an important frequency for detecting noise-induced hearing loss and tinnitus. Our survey was prospectively designed to investigate the prevalence and risk factors of hearing and tinnitus in adolescents. We carefully selected a questionnaire regarding hearing and tinnitus and enrolled subjects in a systematically randomized manner according to region and type of school. All PTA tests were performed at the same facility by a qualified audiologist after an otologic examination by ear-nose-throat physicians. So, our results are representative of tinnitus among South Korean adolescents.

### Prevalence of tinnitus among adolescents

The prevalence of tinnitus among South Korean adolescents is 45.6% and that of severe tinnitus is 9.1%. It is more common in older adolescents and girls. The prevalence of tinnitus among adolescents differs according to the definition, study population, and method of evaluation. [3] The prevalence of tinnitus among adolescents has been reported to be 6.5% [20] to 43.9% [11]. The prevalence of severe tinnitus ranges from 0.6% [15] to 15.72% [16]. The prevalence of transient tinnitus is 39.7% [21] to 73.5% [22]. Prior studies applied different definitions of tinnitus severity, *e*.*g*., bothersome, uncomfortable, worried or concerned, and some used a yes or no response. [23, 24] Here, we used three tinnitus severity categories: no, any, and severe. Severe tinnitus was defined as that which disturbs sleep, a definition that is simple and reliable. [16] Use of the tinnitus handicap inventory or VAS for tinnitus may have yielded more comprehensive data on tinnitus severity. [25]

The prevalence of tinnitus increases with age. [5, 15, 26] However among adolescents, those in their mid-teens have the highest incidence of tinnitus. [23] In this study, older adolescents had a higher prevalence of tinnitus, possibly due to the higher level of stress in high school; however, our use of only two age groups prevents the drawing of any firm conclusions. Among the high school, but not the middle school, students, tinnitus was correlated with academic attainment. The prevalence of tinnitus was higher in females than males, as reported previously. [3, 18, 27] This may be due to the greater tendency of girls to describe symptoms [28] and their more frequent generation of spontaneous otoacoustic emissions [29]. A history of ear infection is reportedly a risk factor for tinnitus and an association between tinnitus and a low household income has been described; [18] our findings were in agreement with these reports and we found that the same factors were associated with hearing loss. [11]

### Factors associated with tinnitus among adolescents

Tinnitus is associated with LNE at karaoke facilities and LAN gaming centers, as well as with a poor HP, but not with hearing loss. Hearing loss is often associated with tinnitus, [6] but not in adolescents. [4, 18] Our data also showed no relationship between tinnitus and hearing loss up to 8 kHz. Noise exposure is a risk factor for tinnitus, but does not induce hearing loss. [18, 22, 27] We found that tinnitus was associated with usage of LAN gaming centers and karaoke facilities, which are noisy leisure activities that many Korean adolescents enjoy. [11] Williams *et al*. also reported that experience of tinnitus is not associated with hearing loss as detected by PTA or otoacoustic emissions (OAEs) in young Australians. [30] However, there was correlation between the experience of tinnitus and the frequency of experience of tinnitus with cumulative life-time noise exposure. [30] An elevated noise level can damage the synapse of the auditory nerve without damaging the outer hair cells. [31] Such damage or hearing loss could induce neuroplasticity in the auditory system and lead to the development of tinnitus. [32] Some patients have a normal hearing threshold by conventional PTA but decreased HP in noisy situations, temporal resolution, and wave I potential in the auditory brain stem response. [9, 32] This is known as hidden hearing loss or cochlear synatopathy. [31, 33] In this study, adolescent patients with tinnitus showed poor HP (poor localization, hearing difficulty in noisy situations, decreased memory for verbal orders, and susceptibility to fatigue). These findings suggest the existence of hidden hearing loss among adolescents. Ultra-high-frequency PTA or other hidden hearing loss tests (*e*.*g*., hearing-in-noise test and auditory brain stem response) may enable detection of hearing deterioration in adolescents. [34] Childhood tinnitus patients are more likely report to deterioration in their hearing ability over time. [4] Patients with tinnitus with normal hearing should wear appropriate ear protection in noisy situations. [27, 30]

### Comorbid conditions of tinnitus among adolescents

Adolescents with tinnitus have a variety of somatization symptoms and frequently smoke cigarettes and consume alcohol. Adolescent patients with tinnitus more often complain about physical health issues, *e*.*g*., severe headache, severe menstrual cramping, throbbing pain in the body, sore throat, digestive problems, and bruising. Attentional mechanism in tinnitus could make this difference. [35] Tinnitus may be associated with somatization and somatoform disorder. [36, 37] Because some conditions, *e*.*g*., pain and headache, have similar pathophysiologic mechanisms they could be symptoms of the same disease. [38, 39]

Adolescents are vulnerable to psycho-emotional factors, which can have detrimental effects on emotional development. [40] Adolescent patients with tinnitus often have mental health problems, such as depression, anxiety, suicidal thoughts, and sleep disturbance. [8, 41, 42] The severity of tinnitus is correlated with psychiatric disorders and the severity of depression and anxiety. [8, 43] Tinnitus can induce psychological symptoms [36], but the causality of the relationship has not been established. In this study, a large proportion of the adolescent patients with tinnitus had a history of bullying or isolation and of carrying a weapon (*e*.*g*., knife or club). In addition, the adolescent patients with tinnitus have a high prevalence of smoking, alcohol drinking, and substance abuse; [5, 18, 19, 44] this is in agreement with our findings. Adolescent are vulnerable to substance abuse, which is common in those with psychiatric comorbidities. [45] Therefore, adolescents with tinnitus should receive appropriate counselling to improve their mental health and prevent substance abuse.

### Limitations

The cross-sectional design of this study prevented assessment of the causality of the associations identified. Smoking and alcohol consumption are reportedly causes of tinnitus. [5, 19, 44] A longitudinal follow up study is needed to assess the causality of the associations and the long-term effect of tinnitus and hearing change in adolescents. This study enrolled students attending middle or high schools. In South Korea, attending middle school is mandated by law and the rate of enrollment in high school was about 93% in 2016. [46] We did not investigate the laterality of tinnitus and objective tinnitus. In addition, testing of tinnitus loudness and pitch matching would have provided further information on tinnitus in adolescents.

## Conclusions

Tinnitus is common among adolescents, particularly females and high-school students. Tinnitus is associated with LNE and educational attainment. Adolescents with tinnitus tend to smoke cigarettes and consume alcohol. Appropriate counseling is needed to alleviate tinnitus and prevent substance abuse. Patients with tinnitus but not hearing loss should wear ear protection in noisy situations to prevent the development of noise-induced hearing loss.

## Supporting information

S1 File Dataset. (XLSX)

S1 Questionnaire Questionnaire for students. (PDF)

S2 Questionnaire Questionnaire for parents. (PDF)

S1 Text (DOCX)

## Abbreviations

PTA: pure tone audiometry
HP: hearing performance
LNE: leisure noise exposure
LAN: local area network
