# Supplementary material for: Prevalence, associated factors, and comorbidities of tinnitus in adolescents
Source: PLoS One. 2020 Jul 31;15(7):e0236723. doi: 10.1371/journal.pone.0236723 (PMC7394400; doi:10.1371/journal.pone.0236723)
Supplement: S1 Text — (DOCX) [file pone.0236723.s004.docx]

The English in this document has been checked by at least two professional editors, both native speakers of English. For a certificate, please see:

http://www.textcheck.com/certificate/AWzt5O
